# Performance of the PointCare NOW System for CD4 Counting in HIV Patients Based on Five Independent Evaluations

**DOI:** 10.1371/journal.pone.0041166

**Published:** 2012-08-09

**Authors:** Michèle Bergeron, Géraldine Daneau, Tao Ding, Nadia E. Sitoe, Larry E. Westerman, Jocelijn Stokx, Ilesh V. Jani, Lindi M. Coetzee, Lesley Scott, Anja De Weggheleire, Luc Boel, Wendy S. Stevens, Deborah K. Glencross, Trevor F. Peter

**Affiliations:** 1 Public Health Agency of Canada, National Laboratory for HIV Immunology, Ottawa, Canada; 2 Immunology Unit, Institute of Tropical Medicine, Antwerp, Belgium; 3 Instituto Nacional da Saúde, Maputo, Mozambique; 4 Division of Global HIV/AIDS, Centers for Disease Control and Prevention, Atlanta, Georgia, United States of America; 5 Clinical Sciences, Institute of Tropical Medicine, Antwerp, Belgium; 6 University of the Witwatersrand, Faculty of Health Science, School of Pathology, Johannesburg, South Africa; 7 Clinton Health Access Initiative, Maputo, Mozambique; Ghent University, Belgium

## Abstract

**Introduction:**

Point-of-care (POC) CD4 testing can improve access to treatment by enabling decentralization and reducing patient loss-to-follow-up. As new POC CD4 technologies become available, their performance should be assessed before widespread deployment. This study reports the findings of five independent evaluations of the PointCare NOW CD4 system.

**Materials/Methods:**

Evaluations were conducted in Southern Africa (Mozambique, South Africa) and North America (Canada, USA). 492 blood samples (55 from HIV-negative blood donors and 437 from HIV-infected patients, including 20 children aged between 12 and 59 months) were tested with both the PointCare NOW and reference flow cytometry instruments. Assessment of bias, precision and levels of clinical misclassification for absolute and percent CD4 count was conducted.

**Results:**

PointCare NOW significantly overestimated CD4 absolute counts with a mean relative bias of +35.0%. Bias was greater in samples with CD4 counts below ≤350cells/µl (+51.3%) than in the CD4 >350cells/µl stratum (15.1%). Bias in CD4% had a similar trend with an overall relative mean bias of +25.6% and a larger bias for low CD4 stratum (+40.2%) than the higher CD4 stratum (+5.8%). Relative bias for CD4% in children was −6.8%. In terms of repeatability, PointCare NOW had a coefficient of variation of 11%. Using a threshold of 350cells/µl, only 47% of patients who qualified for antiretroviral therapy with reference CD4 testing, would have been eligible for treatment with PointCare NOW test results. This was 39% using a 200cells/µl threshold. Agreement with infant samples was higher, with 90% qualifying at a 25% eligibility threshold.

**Conclusion:**

The performance of the PointCare NOW instrument for absolute and percent CD4 enumeration was inadequate for HIV clinical management in adults. In children, the small sample size was not large enough to draw a conclusion. This study also highlights the importance of independent evaluation of new diagnostic technology platforms before deployment.

## Introduction

The HIV epidemic remains a major global public health challenge with an estimated 34 million people living with HIV worldwide [1]. The past decade has witnessed a remarkable global effort to improve access to HIV antiretroviral therapy (ART). Despite the progress, approximately half of all people who need treatment do not yet receive it.

Enumeration of CD4 lymphocytes is an essential diagnostic tool for initiating therapy and monitoring its efficacy [2]. CD4 testing typically relies on complex flow cytometry equipment which requires infrastructure and technical skills which are commonly unavailable at rural and remote clinics [3]. New point-of-care (POC) CD4 technologies enable testing to be decentralized to these sites and for test results to be provided during the course of the patient visit. Recent studies have demonstrated that POC CD4 can significantly improve rates of ART initiation and reduce patient loss-to-follow-up, which is often high before treatment initiation. Immediate access to CD4 results may also enable more rapid initiation of prophylactic treatment for opportunistic infections as well as chemotherapy for prevention of mother-to-child transmission at sites where CD4 levels define the prophylactic drug regimen [4], [5]. Expansion of POC CD4 testing is therefore a priority initiative to improve access to treatment for HIV and AIDS, and several POC CD4 technologies have recently become available, or are expected in the near future [6]–[8]. As these are new technologies with limited track record, rigorous independent assessment of their performance is required so that public health managers can make informed decisions around technology selection and deployment. International prequalification systems for such diagnostic devices are being developed [9].

Introduced in 2008, the PointCare NOW technology (PointCare, Marlborough, MA, USA), was designed for HIV/AIDS patient care in resource-limited settings. This fully automated platform provides absolute CD4 count, CD4% and hematology parameters. To date, no independent evaluation on the performance of PointCare NOW system has been published. This report summarizes results from five independent studies conducted in Southern Africa and North America on the performance of PointCare NOW system for CD4 counting.

## Materials and Methods

### Ethics statement

All studies were approved by respective ethical review committees, and all participants (or guardians for children) provided their written informed consent, except in Johannesburg. In Johannesburg, anonymous samples were used and need for informed consent was waived by the Institutional Review Board of the University of the Witwatersrand (WITS), Johannesburg, South Africa.

### Study Groups

Five sites independently conducted studies to evaluate the performance of the PointCare NOW system for CD4 enumeration. The five evaluating centers were (A) the Instituto Nacional de Saúde (INS) Maputo, Mozambique; (B) the Institute of Tropical Medicine of Antwerp (ITM, Belgium) working in Tete, Mozambique; (C) the Public Health Agency of Canada (PHAC), Ottawa, Canada; (D) the Centers for Disease Control and Prevention (CDC), Atlanta, USA; and (E) WITS, Johannesburg, South Africa. The studies were conducted in 2009 and 2010.

### Blood collection and analysis

Blood samples came primarily from HIV-infected patients. Except for a sub-group of 20 children aged from 12 to 59 months in site B, all were adults. In addition, blood samples from adult HIV-negative blood donors were collected at Site D (Table 1). Whole blood was collected in 2 ml evacuated blood tubes containing EDTA anticoagulant (PointCare, Marlborough, MA, USA or BD Biosciences, Franklin Lakes, NJ, USA) and filled to capacity. Sites B and E conducted a single blood draw for both PointCare NOW and reference instrument testing while the remaining sites used multiple draws with one sample dedicated to the PointCare NOW analysis. When a single tube of blood was drawn, the PointCare NOW analysis was done first using unopened tubes inserted directly into the instrument. All samples were analyzed within 8 hours of collection for PointCare NOW testing and the same day for reference testing, except in site C where 55% of the samples were run the next day but still within 24 hours of collection.

**Table 1:** Table of reference methods and study populations at respective sites.

			Single Platform Reference Method			
Site	N^a^	HIV Status	Instrument	Software	MAb Combo	Beads
A	143^b^	+	FACSCalibur	CellQuest Pro	CD45/3/4/8	TruCount
B	114+20^c^	+	FACSCalibur	CellQuest Pro	CD45/3/4/8	TruCount
C	89	+	FACSCalibur	CellQuest Pro	CD45/3/4/8	FlowCount
D	55^d^	−	FACSCalibur	MultiSet	CD45/3/4	TruCount
E	71	Unknown	Epics-XL	System II	CD45/CD4 FlowCare PLG	FlowCount

a N: number of data sets.

b Site evaluated two PointCare Now with 75 and 68 samples respectively.

c Site recruited 114 adults and 20 children.

d Twenty-seven healthy volunteers were collected. Some were diluted with autologous plasma to obtain low level of CD4 cells for a total of 55 data sets.

### Reference Testing

The reference test method used at each site are listed in Table 1 and consisted of the FACSCalibur instrument (Becton Dickinson Biosciences, San Jose, CA, USA) and the EPICS XL instrument (Beckman Coulter, FL, USA). All employed bead-based single-platform flow cytometry technology with CD45 gating, in accordance with guidelines for performing CD4 enumeration [10]–[12]. Maintenance and instrument calibration was performed according to the respective manufacturer guidelines. Internal quality control was monitored routinely at all sites using stabilized whole blood material. All sites also participated in an external quality assessment (EQA) program. The reference laboratory at site E was accredited by the South African National Accreditation System (SANAS, http://www.sanas.co.za/) and the National Institute of Health (NIH) “Division of Acquired Immunodeficiency Syndrome/Pharmaceutical Product Development” (DAIDS/PPD, http://www.niaid.nih.gov/LabsAndResources/resources/DAIDSClinRsrch/pages/reqnonuslab.aspx) accredited; sites A, B and D participated in and passed the international program for quality assessment and standardization for immunological measures relevant to HIV/AIDS (QASI, http://www.qasi-lymphosite.ca/); and site C was certified by United States National Institute of Allergy and Infectious Diseases (NIAID) CD4 “Immunology Quality Assessment Program” (IQAP, https://iqa.center.duke.edu).

### PointCare Now Testing

The PointCare NOW system (PointCare, Marlborough, MA, USA) is a fully automated closed system which does not require sample preparation. Capped whole blood evacuated blood sample tubes and reagents are inserted directly into the instrument and results are produced in 8 minutes. The technology uses the impedance orifice technique as a white cell counter (WBC) and light-emitting diode (LED) multi-angle light scattering to identify four-part leukocyte differential count. Blood is mixed with anti-CD4 antibody-coated colloidal gold particles which give CD4 lymphocytes a unique refraction signature. Absolute CD4 counts are calculated from WBC, lymphocyte% and CD4% results.

The PointCare NOW system includes a built-in quality control system. The manufacturer recommends to run at least one type of control material. Before performing sample analysis, all sites ran *Daily Check Low and Normal*, a bead suspension control provided by PointCare. In addition, sites A, C and E ran daily fixed whole blood control material *CBCNOW* Low and Normal and *CD4NOW* Low and Normal, also provided by PointCare. All test procedures were conducted following the manufacturer-provided protocols. All sites had a single PointCare NOW machine, except Site A which had two instruments.

### PointCare NOW-reported test failures

The PointCare NOW system has a number of internal checks to validate patient sample analysis which could result in a non-reporting of CD4 results in the case of test failure and a prompt to either re-draw or re-run the sample. In all cases, the tubes were re-run, except on site E which did not record test failures.

### Bias analysis

Bias between CD4 measurements was measured by comparing PointCare NOW test results to the reference method on matched patient samples. Bias was assessed separately for each site, and then overall on raw data combined across sites. Data collected from site A on two instruments was merged into one set. Bland-Altman analysis was carried out to calculate the absolute bias and limits of agreement which are the 95% confidence intervals (±1.96×SD) of the mean bias of all paired measurements in a given category or setting [13]. The Pollock analysis was carried out to calculate the relative bias and limits of agreement which are the 95% confidence intervals (±1.96×SD) of the relative mean bias of all paired measurements in a given category or setting [14]. Percent similarity was calculated for each sample pair as 100*(CD4 on PointCare+CD4 on reference)/(2*CD4 on reference), and median value and coefficient of variation (CV) were calculated per group [15]. Pearson correlation coefficient was also calculated. Differences in parameters between groups were determined by Kruskal-Wallis, Chi-square, or t-test. Statistical analysis was performed with MedCalc statistical software (version 10.0.2.0) and SPSS 12.0. All data were also stratified in two CD4 groups (CD4≤350 and CD4>350 in adults, and CD4%≤25 and CD4%>25 in children), based on the clinically relevant thresholds that the World Health Organization (WHO) recommends for ART initiation [2], [16].

### Within-run precision

In accordance with PointCare NOW user's manual, a single whole blood sample tube cannot be run more than twice in the instrument. Sites C and E assessed PointCare NOW reproducibility on 8 or 10 replicate blood samples from multiple draws from healthy patients taken and measured in parallel. Coefficient of variation (CV%) was calculated for these data sets. Sites A and B ran duplicate patient samples for intra-run variability, and mean percent difference was calculated for each set of CD4 count results. For percent CD4, absolute mean residual values were calculated. Site A also ran duplicate patient samples on two different instruments for inter-run variability (one replicate on each instrument). Reproducibility of the respective reference technology was assessed similarly at all sites on 10 replicate blood samples.

### Determining eligibility for ART

The clinical agreement between PointCare NOW and the reference methods was evaluated based on a threshold for ART initiation of 350 CD4 cells/µl, according to the new WHO guidelines [2]. A 10% bilateral inclusion range was applied, considering values between 332 and 367 as similar. As the previous WHO-recommended cut-off of 200 CD4 cells/µl [17] was still used in some settings, this was also included in the analysis, with a 10% bilateral inclusion range (values from 190 to 210). All adults were included in this analysis, irrespective of their HIV or ART status.

For children, the threshold for ART used was 25% or 750 CD4 cells/µl [16], with a 10% bilateral inclusion range (23.7 to 26.2% for 25, and 712 to 787 cells/µl for 750).

True eligibility for ART was based on the CD4 value obtained from the reference instrument used at each study site. We calculated the sensitivity and specificity, and the *kappa* coefficient for inter-rater agreement [18].

### Compatibility with stabilized blood quality control products

Blood products used for external quality assessment were tested after appropriate preparation in recommended tubes. Reading was performed in both modes “patient” and “control”.

## Results

### PointCare NOW reported test failures

The mean test failure rate on the PointCare NOW instrument was 13.8% (range: 11.0%–18.6%). When samples were re-run, 54% of these failed again. In consequence, no CD4 result was obtained for 9.2% of all samples.

### Bias analysis on absolute CD4 counts in adults

**Figure 1:**
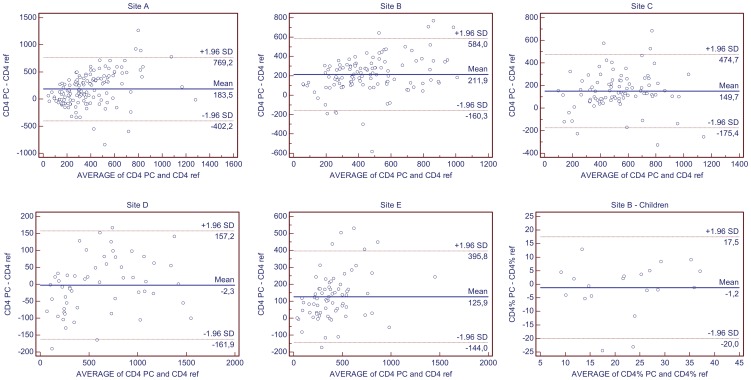
Bland-Altman graphs for CD4 agreement of each site evaluation. Bias was plotted per site (A–E, and for children in site B) on all data range. Horizontal lines report mean bias (Mean) and limits of agreement (1.96 SD). Absolute CD4 count (CD4, in cells/µl), or relative CD4 (CD4%, in %) on PointCare NOW (PC) or on reference instrument (ref).

Overall, the PointCare NOW platform generated higher CD4 counts than the reference method (Table 2) with a median CD4 count of 507 cells/µl and 325 cells/µl, respectively (p<0.0001). Raw data are available in Table S1 agreement data. The mean absolute difference (bias) between methods was +153 cells/µl (95% limits of agreement (LOA): −280 to +586 cells/µl) (Figure 1). This represented a 35% upward bias over the reference technology (LOA: −74.2 to +144.2%). The mean percent bias was higher (+51.3%) for samples with lower CD4 counts (<350cells/µl) than for the high CD4 stratum (+15.1%; p<0.0001). Correlation coefficient and similarity were also significantly different according to CD4 strata (p = 0.0001 and p = 0.015 respectively).

**Table 2:** Bias between reference method and PointCare Now (PC) for CD4 absolute counts.

Lab	CD4 strata	N	Median CD4 cells/µl (Low-High)		p value*	Mean bias (SD)	Mean % bias (SD)	R	Median % Similarity (%CV)
			PC	Reference					
All		472	507 (28–1575)	325 (4–1613)	<0.0001	+153 (221)	+35.0 (55.7)	0.702	119 (111)
	≤350	259	369 (28–1433)	220 (4–350)	<0.0001	+180 (204)	+51.3 (60.9)	0.343	139 (124)
	>350	213	689 (78–1575)	517 (351–1613)	= 0.0006	+120 (236)	+15.1 (40.8)	0.624	110 (21)
A		143	424 (30–1468)	252 (9–1291)	<0.0001	+237 (312)	+43.1 (72.3)	0.384	139 (97)
	≤350	101	386 (30–1433)	194 (9–350)	<0.0001	+226 (252)	+60.4 (67.1)	0.293	162 (96)
	>350	42	562 (78–1468)	459 (353–1291)	= 0.1005	+ 81 (373)	+1.4 (67.8)	0.244	106 (33)
B		114	533 (113–1336)	317 (4–918)	<0.0001	+212 (190)	+51.1 (48.6)	0.710	135 (98)
	≤350	68	450 (113–847)	227 (4–350)	<0.0001	+220 (146)	+67.9 (47.4)	0.566	151 (104)
	>350	46	731 (171–1336)	529 (356–918)	= 0.0027	+200 (241)	+26.3 (38.5)	0.418	117 (19)
C		89	611 (81–1183)	430 (22–1272)	<0.0001	+150 (166)	+31.1 (41.1)	0.743	114 (72)
	≤350	26	405 (81–711)	250 (22–345)	= 0.0013	+181 (163)	+55.2 (57.1)	0.307	139 (102)
	>350	61	670 (122–1183)	506 (350–1272)	= 0.0476	+141 (167)	+34.6 (26.9)	0.626	112 (15)
D		55	527 (28–1498)	541 (109–1613)	<0.0001	−11 (85)	−9.5 (31.2)	0.980	99 (11)
	≤350	21	206 (28–369)	295 (109–342)	= 0.0001	−47 (61)	−27.3 (42.1)	0.739	95 (14)
	>350	34	820 (293–1498)	762 (359–1613)	= 0.0693	+11 (91)	+1.4 (13.9)	0.964	101 (7)
E		71	428 (37–1575)	301 (29–1332)	<0.0001	+126 (138)	+32.0 (37.9)	0.856	118 (31)
	≤350	41	352 (37–739)	221 (29–350)	<0.0001	+124 (115)	+41.8 (41.0)	0.661	128 (14)
	>350	30	596 (202–1575)	469 (356–1332)	= 0.0437	+128 (166)	+18.7 (29.0)	0.798	110 (7)

Comparison in adults for absolute CD4 (CD4), and per group below and above 350 cells/µl.

* paired sample t-test. SD, standard deviation ; CV, coefficient of variation.

**Figure 2:**
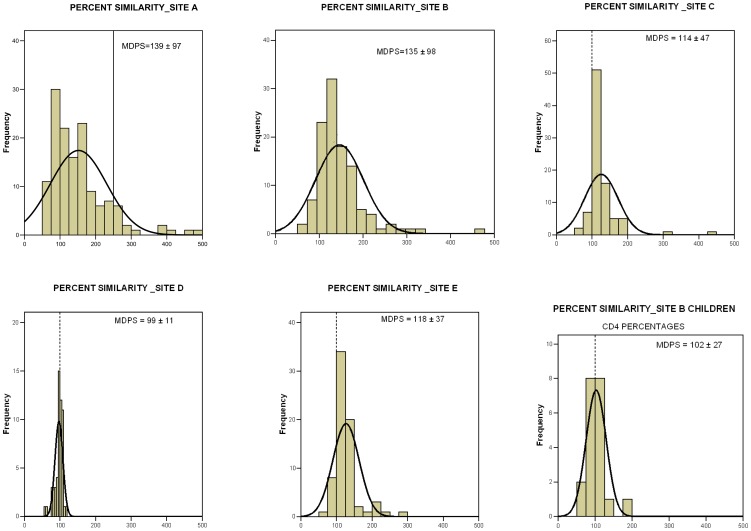
Percent similarity histogram of each site evaluation. Percent similarity values on absolute CD4 counts (Site A–E) or relative CD4 (Site B-Children) were plotted per ranges according to frequency in the respective site. Outlier values corresponding to percent similarity values above 500% were removed: 5 data sets were removed from site A (562, 731, 755, 944, 1572); 2 data sets from site B (704, 1675); and 1 data set from site C (765). For each site, median percent similarity (MDPS) ± standard deviation were calculated.

Agreement between PointCare NOW and reference technology varied by site. Statistical analysis based on individual sites showed larger mean percent bias in the low CD4 ranging from 27.3% to 67.9%. Site D which exclusively analyzed samples from healthy donors reported the best agreement with PointCare NOW with a mean percent bias for both low and high CD4 strata of −27.3% and +1.4% respectively and a median percent similarity (MDPS) close to 100%. The MDPS and SD obtained by the other four sites showed statistically discordant CD4 counts between PointCare NOW and reference methods with a MDPS between 114% and 139% and SD between 37% and 98% (Figure 2).

### Bias analysis on percentage CD4 counts in adults

The performance of PointCare NOW, for percentage CD4 (CD4%) (Table 3), showed a similar trend as for absolute counts with an overall positive absolute bias of +5% (LOA: −16.6 to +26.6%) and a higher absolute bias for the low CD4 level stratum (+7.3%) than for high CD4 stratum (+2.3%, p<0.0001). Median CD4% with PointCare NOW and reference method were 27.1% and 20.0% respectively (p<0.0001). Raw data are available in Table S1 agreement data.

**Table 3:** Bias between reference method and PointCare Now (PC) for CD4 %.

Lab	CD4 strata	N	Median CD4% (Low-High)		P value*	Mean bias (SD)	Mean % bias (SD)	R	Median % Similarity (%CV)
			PC	Reference					
All		472	27.1 (2.4–63.4)	20.0 (0.4–64.0)	<0.0001	+5.0 (11.0)	+24.6 (56.1)	0.614	110 (103)
	≤350	259	23.0 (2.4–50.9)	13.9 (0.4–57.0)	<0.0001	+7.3 (11.7)	+40.2 (63.3)	0.355	126 (116)
	>350	213	32.7 (3.0–63.4)	29.1 (9.0–64.0)	= 0.0006	+2.3 (9.4)	+5.8 (38.3)	0.671	104 (21)
A		143	22.3 (2.4–63.4)	12.0 (1.0–44.0)	<0.0001	+9.0 (12.8)	+43.3 (72.0)	0.196	138 (88)
	≤350	101	21.4 (2.4–49.0)	10.0 (1.0–22.0)	<0.0001	+11.3 (11.7)	+60.0 (68.2)	−0.012	159 (87)
	>350	42	26.2 (3.0–63.4)	21.0 (9.0–44.0)	= 0.1005	+3.6 (13.7)	+3.1 (65.2)	0.255	107 (33)
B		114	25.4 (4.6–54.1)	19.0 (0.4–44.4)	<0.0001	+7.1 (8.8)	+34.5 (49.8)	0.558	121 (120)
	≤350	68	24.3 (4.6–40.5)	15.5 (0.4–30.5)	<0.0001	+9.0 (8.0)	+47.9 (53.5)	0.483	131 (93)
	>350	46	27.6 (6.8–54.1)	23.4 (9.8–44.4)	= 0.0027	+4.3 (9.2)	+14.7 (36.0)	0.553	110 (16)
C		89	32.5 (5.6–63.2)	28.5 (1.8–64.0)	<0.0001	+3.4 (8.5)	+14.1 (39.1)	0.727	103 (53)
	≤350	26	22.8 (5.6–49.7)	18.2 (1.8–31.4)	= 0.0013	+7.4 (9.9)	+37.1 (55.2)	0.436	124 (76)
	>350	61	37.2 (7.1–63.2)	33.9 (11.6–64.0)	= 0.0476	+1.9 (7.3)	+5.2 (25.1)	0.720	103 (13)
D		55	39.4 (4.2–59.1)	43.0 (30.0–58.0)	<0.0001	−6.0 (9.9)	−17.8 (33.4)	0.530	96 (12)
	≤350	21	30.1 (4.2–50.9)	41.0 (31.0–57.0)	= 0.0001	−12.4 (11.1)	−39.8 (42.1)	0.376	89 (14)
	>350	34	43.9 (25.8–59.1)	44.5 (30.0–58.0)	= 0.0693	−2.1 (6.5)	−4.3 (16.0)	0.707	97 (8)
E		71	24.7 (2.5–49.4)	19.1 (2.9–37.2)	<0.0001	+4.1 (7.0)	+17.4 (37.6)	0.713	111 (27)
	≤350	41	19.8 (2.5–44.1)	14.2 (2.9–32.4)	<0.0001	+4.9 (6.5)	+24.0 (41.9)	0.714	114 (14)
	>350	30	28.3 (10.9–49.4)	25.9 (13.3–37.2)	= 0.0437	+3.0 (7.7)	+8.2 (28.8)	0.507	108 (15)

Comparison in adults for relative CD4 (CD4%), and per group below and above 350 cells/µl.

* paired sample t-test. SD, standard deviation ; CV, coefficient of variation.

On a per site basis, each site showed lower bias of PointCare NOW results in the high CD4 stratum than in the low CD4 stratum. Lab D had the largest bias (−12.4%) in the low CD4 stratum, using diluted samples to generate low absolute CD4 counts without affecting CD4% (median CD4% was 41%, for only 10–18% in other sites).

### Bias analysis on infant CD4 counts

Children presented a median CD4% of 24.0% on reference testing, compared to 23.3% on PointCare NOW (Table 4). Raw data are available in Table S2 agreement data children. There was a mean absolute bias of −1.2% (LOA: −20.0 to +17.5%), and a relative bias of −6.8 (LOA: −106.2 to +92.6%). We observed a slight overestimation for the children with less than 25% CD4 (+1.7%), and an underestimation for children with higher CD4% (−4.8%). While the overall difference between PointCare NOW and reference testing was not statistically significant, and percent similarity was close to 100% for both groups, there was low correlation at high CD4% (coefficient = 0.0833). That was partly reflected in large SD for all parameters.

**Table 4:** Bias between reference method and PointCare Now (PC) for children.

	CD4 strata	N	Median CD4% or cells/µl (Low-High)		p-value*	Mean bias (SD)	Mean % bias (SD)	R	Median % Similarity (%CV)
			PC	Reference					
CD4%		20	21.3 (5.3–39.8)	24.0 (7.0–36.6)	= 0.57	−1.2 (9.6)	−6.8 (50.7)	0.537	102 (27)
	≤25%	11	14.4 (8.1–29.5)	16.7 (7.0–24.5)	= 0.32	+1.7 (5.3)	+9.5 (39.5)	0.700	107 (28)
	>25%	9	27.6 (5.3–39.8)	29.9 (25.1–36.6)	= 0.28	−4.8 (12.5)	−26.6 (58.0)	0.0833	97 (22)
CD4		20	1360 (267–3367)	1253 (117–2906)	= 0.49	+107 (673)	+5.6 (56.4)	0.597	113 (29)

Comparison in children for relative (CD4%) for all and per group below and above 25%, and absolute counts in cells/µl (CD4).

* paired sample t-test. SD, standard deviation ; CV, coefficient of variation.

Median absolute CD4 counts were 1253 cells/µl on reference, and 1360 cells/µl on PointCare NOW, giving an absolute bias of +107 cells/µl (LOA: −1212 to +1425 cells/µl), and a relative bias of +5.6% (LOA: −104.9 to +116.2%).

### Within-run precision

Precision measurements of PointCare NOW and the reference technologies are presented in Table 5. Raw data are available in Table S3 precision data. As per supplier specification, percent CV on the PointCare NOW instrument is expected to be less than 10 to 15% across high and low CD4 counts respectively. PointCare NOW precision at sites C, D and E were all below 11%.

**Table 5:** Within run precision assessment for absolute CD4 count (cells/µl) and relative CD4 measurement (%).

Site	Reference method		PointCare Now	
	CD4%	CD4 cells/µl	CD4 %	CD4 cells/µl
A	3.6% (18.7±0.7)	5.0% (429±21.6)	n/a	n/a
B	2.2% (40.7±0.9)	3.8% (806±30.6)	n/a	n/a
C	1.7% (38.6±0.6)	3.4% (795±26.7)	9.6%* (47.7±4.6)	10.6%* (798±84.4)
D	1.2% (53±0.6)	3.7% (1089±40.3)	4.4% (50.2±2.2)	5.1% (1108±57)
E	1.4% (51.4±0.7)	4.0% (1456±58.2 )	3.9% (50.4±2.0)	5.1% (1405±71.7)

Intra-run precision given as %CV (Mean ± SD) on 10 replicates.

* PointCare Now precision established from 8 replicates.

At the two sites which assessed repeatability based on duplicates, mean percent differences between duplicate CD4 counts were 39% for site A (4 sets of duplicates) and 17% for site B (3 sets of duplicates). Mean absolute difference in CD4% was 7% in site A and 3% in site B. The inter-instrument variability based on 8 duplicates in site A demonstrated a mean percent difference of 70% for absolute counts, and a 20% absolute difference in CD4%.

The coefficients of variation for all reference technologies were equal to or lower than 5%.

### Determining eligibility for ART

The clinical agreement between PointCare NOW and the reference methods was evaluated based on different decisional threshold to initiate ART. Using a 350 CD4 cells/µl threshold, sensitivity for detection of treatment eligibility by PointCare NOW amongst adults was overall 53%, and less than 65% in all study sites using patient blood samples (Table 6). Patients with ≤350 CD4 cells/µl on reference instrument (i.e. treatment eligible) represented 53% of all patients, hence approximately 50% of ART-eligible patients were misclassified as ineligible by PointCare NOW. The average overestimation of CD4 levels (min-max) in the 119 misclassified patients was 323 cells/µl (79–1264). Specificity of PointCare NOW was overall 94%, and always higher than 90%, with the exception of site A.

**Table 6:** Clinical agreement between PointCare NOW and Reference method at 2010 WHO guidelines in adults.

Sites	N	N below cut-off	Sensitivity	Specificity	*kappa* coefficient
A	143	98	49%	80%	0.233
B	114	65	38%	96%	0.314
C	89	28	50%	100%	0.578
D	55	20	100%	97%	0.971
E	71	40	63%	94%	0.535
All	472	251	53%	94%	0.450

Case identification at cut-off of 350 cells/µl is based on theoretical decision to initiate ART based on PointCare NOW values to decision based on the reference instrument, with a 10% bilateral inclusion range (values from 332 to 367 were considered as similar).

Using an ART threshold of 200 CD4 cells/µl, 22% of the overall study population were treatment eligible by reference testing. PointCare NOW correctly classified eligibility in 39% of these patients (Table 7). The average overestimation of CD4 levels in the 65 misclassified patients was 302 cells/µl. The specificity with the 200 CD4 cells/µl threshold was 94%.

**Table 7:** Clinical agreement between PointCare NOW and Reference method at 2006 WHO guidelines for adults.

Sites	N	N below cut-off	Sensitivity	Specificity	*kappa* coefficient
A	143	50	32%	85%	0.187
B	114	23	26%	96%	0.275
C	89	9	44%	98%	0.492
D	55	6	100%	94%	0.770
E	71	18	50%	100%	0.599
All	472	106	39%	94%	0.377

Case identification at cut-off of 200 cells/µl is based on theoretical decision to initiate ART based on PointCare NOW values to decision based on the reference instrument, with a 10% bilateral inclusion range (values from 190 to 210 were considered as similar).

Sensitivity and specificity to identify children in need of ART using a 25% CD4 threshold were 90% and 70% respectively, and 100% and 88% using a 750 CD4 cells/µl threshold (Table 8).

**Table 8:** Clinical agreement between PointCare NOW and Reference method at 2010 WHO guidelines for children.

Cut-off	N	N below cut-off	Sensitivity	Specificity	*kappa* coefficient
25%	20	10	90%	70%	0.600
750 cells/µl	20	3	100%	88%	0.692

Case identification at cut-off of 25% or 750 cells/µl is based on theoretical decision to initiate ART based on PointCare NOW values to decision based on the reference instrument, with a 10% bilateral inclusion range (values from 23.7 to 26.2 were considered as similar to 25%, and 712 to 787 to 750cells/µl).

### Compatility with stabilized blood quality control products

A few sites made attempt to incorporate stabilized whole blood products. None of the products tested were compatible with PointCare NOW which means that the system failed to give CD4 measurements when run in the patient mode. Products tested included, in Ottawa: Immuno-Trol™ (Beckman Coulter, US); CD-Chex® Plus, CD-Chex Plus® BC, CD4 Count (Streck, US); BD Multi-Check CD4 Control, BD Multi-Check CD4 low Control (BD BioSciences, US); StatusFlow®, (R&D Systems Hematology, US); CytoFix CD4, (Cytomark, UK). Immuno-Trol™ (Beckman Coulter, US) and African Regional External Quality Assessment (AFREQUAS) controls also failed in Johannesburg. Only CD-Chex® Plus (Streck, US) could be read using the control mode.

## Discussion

Enumeration of CD4 T lymphocyte plays a critical role in clinical management of HIV/AIDS patients for initiating and monitoring therapy. It is therefore important that CD4 counts be reliable and precise for optimal patient care. This study demonstrated that absolute and percentage CD4 count test results obtained on adult samples with the PointCare NOW platform did not agree closely with results produced from matched samples tested with reference BD FACSCalibur, and Beckman Coulter EPICS platforms. There was significant positive bias contributing to the low sensitivity of the PointCare NOW system for identifying ART-eligible adults, at both the 200 and 350 cells/µl thresholds. Clinical agreement for tests conducted on children was acceptable albeit the sample size was too small to draw definitive conclusions.

Point-of-care CD4 devices are expected to play a significant role in the scaling up of HIV treatment and care, by reducing the infrastructure and operator skill requirements for testing thereby enabling the further decentralization of CD4 testing, and by reducing test result turn-around times and patient travel and other costs thereby improving pre-ART retention and ART initiation rates [19]–[21]. Miniaturization or less proven test methods that are not based on the established flow cytometry standard may compromise the performance of point-of-care CD4 technologies. Some compromise may be permissible if weighed against the public health benefits provided it does significantly affect clinical decision-making around the initiation or modification of treatment. In this study, 50% of patients in need of ART would not have received treatment based on the PointCare NOW test results when compared to reference testing. While a small bias might not affect patient outcome significantly and might still enable clinical judgment to appropriately manage patients and limit delays in treatment initiation, larger biases can place patients in different clinical categories, affect the level of examination, care or follow-up, and place patients at greater unnecessary risk of morbidity and mortality.

PointCare NOW is a fully automated system which integrates desirable features such as cap piercing technology which eliminates pipetting and reduces biohazard exposure associated with infectious specimens. Process checks to alert user of potential problems during sample analysis are also a desirable feature of this platform. However, the frequency of failure to produce results, even after retesting was high (9.2%), was an important concern from a cost-efficiency and patient wait-time perspective.

The precision of the PointCare NOW instrument on 3 of the 5 sites testing 8–10 replicates was acceptable. However, duplicate testing using HIV-infected blood samples, performed at the two remaining sites, indicated poor reliability of testing.

The factors leading to the overall poor agreement between PointCare NOW and the reference methods in sites using patient blood samples are unclear; however concern lies with the inability of the instrument's built-in quality control to depict system failure considering that controls ran at each site passed daily quality control runs. The time delay between measurements on the PointCare NOW and reference instrument for some samples is unlikely to explain the bias as the majority of tests were conducted on the same day. Furthermore, all samples tested on the reference instruments the day after collection were evaluated using a gating strategy which has been proven accurate on samples up to 4 days after blood draw [11], [22].

The better performance of PointCare NOW in site D may be related to the exclusive use of blood from HIV uninfected individuals and the dilution of blood sample to achieve low CD4 level. It is conceivable that reduced cell density resulting from diluted blood samples may play a role in the binding between gold particles and CD4 lymphocytes cluster which could improve the resolution of the CD4 lymphocyte cluster and overall accuracy of the measurement. Nevertheless, the inclusion of this data set does not alter the qualitative results of the study which raise concern about the ability of the instrument to provide accurate and reproducible values with samples from HIV patients.

PointCare NOW technology incompatibility with commonly used external quality controls is a major drawback, as quality assessment is a main factor in implementing decentralized testing [23].

This study also highlights the importance of instrument evaluation independent of the manufacturers, to inform potential users, especially where diagnostic regulatory systems are under development. For those health facilities currently performing CD4 enumeration on PointCare NOW instruments, awareness of the potential for bias with this instrument is recommended. Furthermore, the inaccuracy may be variable between samples and cannot be systematically corrected.

In conclusion, due to the overestimation of both CD4 absolute and percentage count, CD4 enumeration using PointCare NOW resulted in significant misclassification of ART-eligibility and potential “under treatment” of eligible HIV patients, especially in adults.

While they are similar, it is not clear if the new HumaCount CD4^now^ (Human Diagnostics Worldwide, Wiesbaden, Germany) is the same instrument as the PointCare NOW.

## Supporting Information

Table S1 Agreement data. Raw data from all study sites on reference and PointCare instruments, for CD4 counts and CD4%.
(PDF)

Table S2 Agreement data children. Raw data from children in Tete study site on reference and PointCare instruments, for CD4 counts and CD4%, and with age (in months).
(XLS)

Table S3 Precision data. Raw data for replicate measurements in all study sites on reference and PointCare instruments, for CD4 counts and CD4%.
(XLS)

## References

[1] World Health Organization (2011) Global HIV/AIDS response: Epidemic update and health sector progress towards universal access. Progress report 2011. Available: http://whqlibdoc.who.int/publications/2011/9789241502986_eng.pdf. Accessed 2012 Jun 26.

[2] World Health Organization (2010) Antiretroviral therapy for HIV infection in adults and adolescents: recommendations for a public health approach: 2010 revision. Available: http://whqlibdoc.who.int/publications/2010/9789241599764_eng.pdf. Accessed 2012 Jun 26. 23741771

[3] Mandy F, Janossy G, Bergeron M, Pilon R, Faucher S (2008) Affordable CD4 T-cell enumeration for resource-limited regions: a status report for 2008. Cytometry B Clin Cytom 74 Suppl 1: S27–S39 doi: 10.1002/cyto.b.20414. 18307251 10.1002/cyto.b.20414

[4] Lynen L, Thai S, De MP, Leang B, Sokkab A, et al (2006) The added value of a CD4 count to identify patients eligible for highly active antiretroviral therapy among HIV-positive adults in Cambodia. J Acquir Immune Defic Syndr 42: 322–324 doi: 10.1097/01.qai.0000221682.37316.d5. 16688095 10.1097/01.qai.0000221682.37316.d5

[5] Miiro G, Nakubulwa S, Watera C, Munderi P, Floyd S, et al (2010) Evaluation of affordable screening markers to detect CD4+ T-cell counts below 200 cells/µl among HIV-1-infected Ugandan adults. Trop Med Int Health 15: 396–404 TMI2471 [pii].doi: 10.1111/j.1365-3156.2010.02471.x. 20180936 10.1111/j.1365-3156.2010.02471.x

[6] Jani IV, Sitoe NE, Chongo PL, Alfai ER, Quevedo JI, et al (2011) Accurate CD4 T-cell enumeration and antiretroviral drug toxicity monitoring in primary healthcare clinics using point-of-care testing. AIDS 25: 807–812 doi: 10.1097/QAD.0b013e328344f424. 21378535 10.1097/QAD.0b013e328344f424

[7] World Health Organization (2010) Adapting WHO normative HIV guidelines for national programmes: essential principles and processes. Available: http://whqlibdoc.who.int/publications/2011/9789241501828_eng.pdf. Accessed 2012 Jun 26.

[8] Murtagh MM (2011) HIV/AIDS. Diagnostic Landscape. UNITAID Technical Report. Available: http://www.unitaid.eu/images/marketdynamics/publications/unitaid_md_technical_report_diagnostics_landscape_web.pdf. Accessed 2012 Jun 26.

[9] Peter TF, Shimada Y, Freeman RR, Ncube BN, Khine AA, et al (2009) The need for standardization in laboratory networks. Am J Clin Pathol 131: 867–874 131/6/867 [pii]. doi: 10.1309/AJCPCBMOHM7SM3PJ. 19461096 10.1309/AJCPCBMOHM7SM3PJ

[10] Denny TN, Gelman R, Bergeron M, Landay A, Lam L, et al (2008) A North American multilaboratory study of CD4 counts using flow cytometric panLeukogating (PLG): a NIAID-DAIDS Immunology Quality Assessment Program Study. Cytometry B Clin Cytom 74 Suppl 1: S52–S64 doi: 10.1002/cyto.b.20417. 18351622 10.1002/cyto.b.20417

[11] Glencross DK, Janossy G, Coetzee LM, Lawrie D, Aggett HM, et al (2008) Large-scale affordable PanLeucogated CD4+ testing with proactive internal and external quality assessment: in support of the South African national comprehensive care, treatment and management programme for HIV and AIDS. Cytometry B Clin Cytom 74 Suppl 1: S40–S51 doi: 10.1002/cyto.b.20384. 18228554 10.1002/cyto.b.20384

[12] Mandy FF, Nicholson JK, McDougal JS (2003) Guidelines for performing single-platform absolute CD4+ T-cell determinations with CD45 gating for persons infected with human immunodeficiency virus. Centers for Disease Control and Prevention. MMWR Recomm Rep 52: 1–13. 12583540

[13] Bland JM, Altman DG (1999) Measuring agreement in method comparison studies. Stat Methods Med Res 8: 135–160. 10501650 10.1177/096228029900800204

[14] Pollock MA, Jefferson SG, Kane JW, Lomax K, MacKinnon G, et al (1992) Method comparison–a different approach. Ann Clin Biochem 29 (Pt 5) 556–560. 1444169 10.1177/000456329202900512

[15] Scott LE, Galpin JS, Glencross DK (2003) Multiple method comparison: statistical model using percentage similarity. Cytometry B Clin Cytom 54: 46–53 doi: 10.1002/cyto.b.10016. 12827667 10.1002/cyto.b.10016

[16] World Health Organization (2010) Antiretroviral therapy for HIV infection in infants and children: Towards universal access. Recommendations for a public health approach: 2010 revision. Available: http://whqlibdoc.who.int/publications/2010/9789241599801_eng.pdf. Accessed 2012 Jun 26. 23741772

[17] World Health Organization (2006) Antiretroviral therapy for HIV infection in adults and adolescents: recommendations for a public health approach (2006 revision). Available: http://www.who.int/hiv/pub/guidelines/artadultguidelines.pdf. Accessed 2012 Jun 26. 23741771

[18] Cohen J (1960) A coefficient of agreement for nominal scales. Educational and Psychological Measurement 20: 37–46.

[19] Jani IV, Sitoe NE, Alfai ER, Chongo PL, Quevedo JI, et al (2011) Effect of point-of-care CD4 cell count tests on retention of patients and rates of antiretroviral therapy initiation in primary health clinics: an observational cohort study. Lancet 378: 1572–1579 S0140-6736(11)61052-0 [pii];doi: 10.1016/S0140-6736(11)61052-0. 21951656 10.1016/S0140-6736(11)61052-0

[20] Faal M, Naidoo N, Glencross DK, Venter WD, Osih R (2011) Providing immediate CD4 count results at HIV testing improves ART initiation. J Acquir Immune Defic Syndr 58: e54–e59 doi: 10.1097/QAI.0b013e3182303921. 21857356 10.1097/QAI.0b013e3182303921

[21] Peterson K, van Grinsven J, Huis in 't Veld D, Colebunders R (2012) Interventions to reduce mortality in sub-Saharan Africa among HIV-infected adults not yet on antiretroviral therapy. Expert Rev Anti Infect Ther 10: 43–50 doi: 10.1586/eri.11.151. 22149613 10.1586/eri.11.151

[22] Bergeron M, Nicholson JK, Phaneuf S, Ding T, Soucy N, et al (2002) Selection of lymphocyte gating protocol has an impact on the level of reliability of T-cell subsets in aging specimens. Cytometry 50: 53–61 doi: 10.1002/cyto.10092. 12116346 10.1002/cyto.10092

[23] Shott JP, Galiwango RM, Reynolds SJ (2012) A Quality Management Approach to Implementing Point-of-Care Technologies for HIV Diagnosis and Monitoring in Sub-Saharan Africa. J Trop Med 2012: 651927 doi: 10.1155/2012/651927. 22287974 10.1155/2012/651927 PMC3263631

