# Correction: Performance of the PointCare NOW System for CD4 Counting in HIV Patients Based on Five Independent Evaluations

**DOI:** 10.1371/annotation/71a6d947-c034-4749-a33d-971284ea3408

**Published:** 2012-09-19

**Authors:** Michèle Bergeron, Géraldine Daneau, Tao Ding, Nadia E. Sitoe, Larry E. Westerman, Jocelijn Stokx, Ilesh V. Jani, Lindi M. Coetzee, Lesley Scott, Anja De Weggheleire, Luc Boel, Wendy S. Stevens, Deborah K. Glencross, Trevor F. Peter

There is an error in the Competing interests statement. The correct statement is: The SA NHLS is the sole owner of the PLG CD4 patent, and receives royalties related to licensing agreement with BC Miami. 

