# Supplementary material for: Performance of the PointCare NOW System for CD4 Counting in HIV Patients Based on Five Independent Evaluations
Source: PLoS One. 2012 Aug 9;7(8):e41166. doi: 10.1371/journal.pone.0041166 (PMC3415398; doi:10.1371/journal.pone.0041166)
Supplement: Table S1 — Agreement data. Raw data from all study sites on reference and PointCare instruments, for CD4 counts and CD4%. (PDF) [file pone.0041166.s001.pdf]

ALL SITES

CDC

Count
55

Sample ID	CD4 Percent Measurement		CD4 Absolute Count	
	PointCare METHOD A	Reference Method	PointCare METHOD A	Reference Method
2009490285	49.1	53.5	1064	1129
2009490286	50.2	53.2	1108	1089
2009490287	45.6	46.0	1421	1421
2009490288	40.2	36.8	1451	1278
2009490289	34.1	30.0	699	633
2009490290	32.7	44.0	225	237
2009490291	35.8	42.0	299	299
2009490292	37.9	44.0	198	193
2009490293	44.4	44.0	965	999
2009490294	30.1	32.0	199	173
2009490295	53.4	56.0	1092	1152
2009490296	41.0	58.0	347	416
2009490299	52.4	55.0	1151	1184
2009490300	43.9	56.0	242	272
2009490301	34.5	57.0	206	302
2009490302	43.5	49.0	865	930
2009490304	21.2	51.0	134	229
2009490305	41.8	41.0	713	662
2009490306	24.8	43.0	186	312
2009490307	27.1	40.0	211	301
2009490308	4.2	44.0	28	216
2009490309	21.1	37.0	99	142
2009490310	29.7	45.0	504	707
2009490311	18.7	37.0	200	342
2009490312	40.5	44.0	1371	1382
2009490313	44.2	38.0	825	695
2009490314	49.8	55.0	1441	1492
2009490315	44.1	53.0	976	1067
2009490316	39.4	30.0	470	359
2009490317	44.0	40.0	815	708
2009490318	59.1	53.0	689	554
2009490319	26.8	31.0	106	109
2009490320	32.4	40.0	245	295
2009490321	50.9	53.0	298	296
2009490322	41.9	40.0	527	468
2009490323	38.0	39.0	165	144
2009490324	48.5	52.0	1002	1066
2009490325	44.9	51.0	498	489
2009490326	38.3	41.0	862	819
2009490327	25.8	43.0	293	427
2009490328	47.7	52.0	1200	1093
2009490330	43.7	38.0	791	667
2009490331	33.0	38.0	314	338
2009490332	34.0	39.0	615	635
2009490333	23.8	41.0	212	297
2009490334	51.2	58.0	1498	1613
2009490335	49.8	57.0	749	747

	2009490336	32.2	30.0	594	541
	2009490337	27.2	32.0	292	299
	2009490338	51.5	45.0	798	696
	2009490339	50.0	45.0	369	334
	2009490340	33.3	30.0	992	854
	2009490341	31.0	31.0	469	428
	2009490342	36.5	40.0	690	776
	2009490343	9.7	39.0	30	111
**PHAC**	23378	37.2	39.3	1015	914
	23397	37.6	35.7	799	670
**Count**	23398	63.2	64.0	648	977
**89**	23415	37.0	29.2	573	387
	23416	32.0	30.1	829	713
	23429*	38.3	37.5	552	453
	23430*	32.5	33.7	553	466
	23431*	41.9	40.3	880	618
	23434	45.2	45.4	779	651
	23435	30.2	23.4	437	291
	23436	44.2	41.0	923	705
	23456	49.7	30.0	665	323
	23457	46.7	39.6	721	462
	23458	39.0	26.7	988	593
	23459	30.8	31.1	454	376
	23479	40.1	37.6	766	616
	23481	37.8	32.9	718	541
	23483	47.7	44.7	851	588
	23484	47.6	38.2	586	373
	23485	35.3	22.3	1024	499
	23487*	30.8	27.5	546	392
	23488*	42.0	40.5	622	485
	23514	20.6	22.2	971	867
	23515*	7.1	28.1	122	351
	23516*	30.9	23.4	663	418
	23518	32.7	24.1	694	407
	23521	49.5	25.8	1113	430
	23522	36.8	36.9	852	651
	23523	16.7	4.4	175	22
	23525	21.3	25.2	414	378
	23526*	28.1	15.1	685	281
	23527	22.9	18.3	613	366
	23528	28.9	23.3	670	427
	23565*	34.9	36.7	646	517
	23566	45.6	50.8	940	788
	23567	29.1	29.0	634	444
	23568	38.2	20.7	578	211
	23569	30.9	33.9	654	544
	23583	51.2	24.4	885	599
	23584*	24.9	24.3	367	291
	23585	37.5	35.6	623	500
	23586	17.6	1.8	345	24
	23587	22.5	12.5	552	255
	23588	22.8	14.6	760	409
	23605	11.7	8.2	338	178
	23625	35.7	7.8	711	138

	23626	47.8	21.8	658	235
	23627	45.3	42.0	1183	887
	23630	25.3	43.1	441	400
	23631*	41.9	54.6	887	1026
	23632	32.9	33.8	828	726
	23653*	37.5	35.0	567	360
	23654*	41.3	39.6	506	678
	23655*	42.6	41.1	931	467
	23656*	42.4	39.6	648	760
	23775*	18.7	19.3	352	296
	23778*	26.8	25.3	357	296
	23779	25.9	26.9	511	444
	23780	37.9	32.8	716	505
	23781	39.9	38.9	531	436
	23782	11.9	11.6	422	366
	23794	28.8	26.6	484	392
	23795*	49.3	42.9	597	465
	23796	28.9	26.5	611	523
	23797	19.3	10.4	190	77
	23798	14.8	30.8	139	256
	23817	30.9	26.2	799	671
	23840	30.9	20.0	404	247
	C290	44.6	40.9	787	610
	23844	27.8	25.2	445	364
	23845	20.1	13.2	322	206
	23847	35.0	34.9	857	739
	23848	27.6	28.5	415	345
	23870	37.2	41.3	565	508
	23872	22.8	10.2	389	150
	23873	37.3	30.7	678	506
	23890	32.1	32.5	578	495
	23891	17.2	18.1	560	528
	23897	21.4	10.3	460	169
	23898	5.6	17.0	81	204
	23899	19.5	15.8	492	268
	23900	33.3	36.0	707	603
	23901	37.3	31.4	406	308
	23902	22.8	21.9	407	325
	23919	45.2	55.8	1016	1272
	23920	23.0	16.6	434	252
	23921	21.9	22.0	217	210
	23949	13.5	18.9	154	196
	23950	28.2	27.7	363	347
**South A.**	5170306	31.5	27.7	389	281
	5170271	28.0	23.4	654	392
Count	5170218	28.4	18.4	475	266
71	5170291	29.6	14.6	352	110
	5170367	24.7	15.3	447	218
	5170213	28.3	19.3	528	294
	5170372	11.1	8.1	295	155
	5170287	21.1	9.3	618	181
	5174219	19.9	18.8	550	466
	5174227	20.6	23.2	249	225
	5174236	33.7	13.3	739	236

5174211	28.3	22.0	500	350
5174216	16.8	10.4	407	239
5174090	13.6	13.3	574	510
5174094	36.8	27.9	739	455
5174175	11.8	13.4	255	224
5181532	44.1	22.3	428	116
5181530	31.4	18.5	1091	642
5181535	36.6	26.1	933	528
5181538	28.8	25.5	537	465
5185836	25.6	31.7	370	374
5185913	2.5	2.9	37	37
5185872	45.1	21.4	885	356
5185713	32.7	31.6	478	422
5185785	5.6	12.6	48	56
5184798	2.8	9.4	44	128
5185362	17.3	18.4	274	261
5185068	13.2	13.0	293	262
5185145	28.1	26.6	405	302
5185320	10.9	19.0	271	387
5189779	27.0	21.3	390	290
5189675	12.6	12.8	360	350
5189566	13.0	25.7	202	377
5189603	25.6	22.4	570	433
5189627	30.9	26.6	550	379
5189653	21.4	17.5	427	301
5189696	20.3	19.4	360	302
5189751	15.7	16.7	182	150
5190340	15.8	10.9	367	221
5193124	28.5	33.5	382	394
5193067	27.3	27.9	957	1012
5193309	37.8	32.4	1575	1332
5193085	13.4	6.2	147	56
5193169	19.9	12.8	457	275
5193118	30.9	30.6	454	411
5198193	26.9	21.7	470	327
5198238	24.1	13.9	360	188
5198325	13.8	11.3	283	209
5198229	10.6	5.5	350	177
5198250	35.4	37.2	821	794
5200129	27.0	23.1	447	359
5200143	49.4	30.8	799	490
5200145	22.2	19.1	744	728
5200238	40.5	33.5	611	475
5204218	16.3	12.5	250	168
5203953	22.2	17.4	465	297
5204134	27.3	21.6	580	382
5204155	32.1	29.1	834	690
5203966	21.4	17.3	646	477
5204184	43.0	32.4	115	71
5204187	17.5	14.2	298	184
5204175	19.8	13.6	275	153
5203778	18.0	24.5	248	253
5209622	27.3	21.5	630	472
5209566	14.5	17.1	203	199

	5209587	27.7	33.6	543	533
	5209514	30.1	18.6	494	277
	5209557	16.3	9.8	200	119
	5209523	18.4	27.7	405	516
	5209655	15.9	3.4	145	29
	5209484	33.3	25.3	938	673
**TETE**	903001	22.9	**19.8**	304	**196**
	903002	37.5	**32.7**	1103	**585**
**Count**	903048	39.3	**21.3**	480	216
**114**	903049	24.3	**15.1**	455	287
	903050	33.8	**20.0**	684	296
	903052	34.4	**14.7**	488	173
	903053	25.1	**25.3**	261	188
	903054	29.3	**16.0**	794	326
	903056	33.9	**33.1**	707	457
	903057	18.7	**14.3**	420	285
	903058	23.7	**23.1**	402	303
	903059	34.8	**15.9**	445	163
	903061	30.7	**28.0**	482	338
	903062	25.0	**20.4**	973	710
	903063	17.6	**12.9**	358	196
	903065	27.4	**21.3**	933	579
	903069	39.1	**25.6**	1245	479
	903070	42.5	**36.2**	978	604
	1003004	28.3	**24.8**	1105	763
	1003005	51.8	**41.9**	1131	768
	1003006	34.0	**22.6**	389	210
	1003007	20.1	**8.8**	455	100
	1003008	31.3	**9.1**	345	84
	1003009	28.7	**20.8**	585	327
	1003010	25.3	**11.3**	668	233
	1003011	26.7	**16.3**	602	375
	1003012	25.4	**16.3**	624	350
	1003013	8.4	**13.3**	277	578
	1003014	39.1	**24.0**	644	317
	1003015	13.4	**9.2**	349	262
	1003017	26.2	**23.7**	561	391
	1003018	34.8	**33.3**	785	660
	1003019	19.8	**9.8**	680	399
	1103001	23.5	**17.8**	343	223
	1103003	10.2	**23.5**	172	346
	1103005	29.2	**30.7**	575	462
	1103006	12.0	**7.9**	530	300
	1103007	30.6	**25.8**	642	508
	1103008	40.5	**17.4**	133	37
	1103009	42.0	**43.9**	817	776
	1103010	16.0	**12.3**	644	364
	1103011	27.0	**17.9**	479	264
	1103012	30.0	**29.9**	701	540
	1103013	41.8	**29.1**	1038	685
	1503001	29.6	**19.0**	347	264
	1503002	30.8	**32.4**	555	635
	1503004	19.4	**10.2**	338	174
	1503005	28.3	**15.9**	456	266

1503006	22.3	**20.4**	532	454
1503007	19.9	**22.5**	534	625
1503008	23.6	**18.3**	482	374
1503009	13.9	**9.9**	238	162
1503010	25.1	**11.7**	549	223
1503011	20.3	**20.1**	550	274
1503012	24.2	**7.2**	442	130
1503013	21.5	**17.7**	728	673
1503017	34.8	**26.1**	425	287
1503018	7.5	**16.3**	171	356
1503019	47.6	**30.0**	825	518
1503020	27.8	**19.4**	617	407
1503021	23.5	**16.7**	684	487
1503022	20.0	**1.9**	115	9
1503023	16.1	**6.1**	420	150
1503024	16.3	**3.2**	158	28
1503025	22.8	**11.1**	531	230
1503026	33.7	**9.0**	847	205
1503027	30.9	**33.2**	960	833
1503028	24.4	**13.6**	580	370
1503029	54.1	**28.4**	1336	635
1503030	46.1	**23.2**	939	439
1503031	28.9	**18.0**	457	270
1503061	32.5	**19.5**	579	303
1603001	37.9	**23.5**	583	316
1603002	23.1	**15.9**	734	392
1603003	23.2	**12.0**	765	323
1603004	17.5	**9.6**	746	311
1603006	23.1	**20.5**	807	334
1603007	17.7	**4.1**	372	74
1603008	28.5	**19.1**	348	192
1603009	32.5	**5.0**	345	42
1603010	32.7	**32.7**	925	769
1603011	20.8	**21.6**	857	737
1603012	4.9	**9.3**	144	114
1603014	28.0	**13.8**	537	220
1603015	20.6	**7.3**	528	158
1603016	10.5	**0.4**	116	4
1603017	7.5	**17.4**	134	168
1603018	20.5	**6.6**	312	83
1603019	17.7	**11.5**	643	361
1603020	32.8	**31.4**	651	512
1603021	4.6	**17.4**	113	304
1603025	34.7	**30.0**	1100	918
1703001	23.5	**20.2**	334	258
1703002	18.1	**7.8**	531	231
1703004	18.4	**16.3**	507	434
1703005	25.9	**26.4**	734	681
1703006	24.5	**14.0**	512	274
1703007	21.2	**8.4**	395	136
1703008	5.7	**9.9**	147	247
1703009	24.9	**19.2**	631	517
1703010	25.4	**24.5**	419	306
1703011	15.9	**5.8**	312	123

	1703012	26.6	**21.2**	781	702
	1703013	24.3	**19.4**	964	824
	1803001	33.7	**19.9**	500	201
	1803003	28.8	**30.5**	493	318
	1803004	36.1	**20.1**	578	182
	1803005	6.8	**44.4**	192	777
	1803006	33.8	**20.3**	1185	478
	1803009	28.0	**26.1**	519	325
	1803011	23.1	**14.6**	496	206
	1803016	36.3	**26.1**	958	499
	1803017	28.0	**20.1**	715	343
	2604004	11.2	11.6	356	167
**Maputo**	PC-1-020	15.9	12.0	199	112
**PC-A**	PC-1-007	22.3	10.0	677	304
**Count**	PC-1-009	20.1	10.0	474	212
**75**	PC-1-012	16.9	20.0	275	456
	PC-1-013	39.3	12.0	825	224
	PC-1-015	39.3	18.0	484	186
	PC-1-017	36.8	15.0	353	283
	PC-1-001	63.4	44.0	1279	1057
	PC-1-030	22.6	31.0	327	417
	PC-1-040	31.5	2.0	304	17
	PC-1-029	22.9	16.0	376	244
	PC-1-028	34.4	11.0	1041	331
	PC-1-026	19.7	1.0	274	9
	PC-1-024	20.7	19.0	386	307
	PC-1-022	24.6	10.0	428	153
	PC-1-034	15.4	14.0	277	181
	PC-1-032	23.4	9.0	486	191
	PC-1-062	38.8	11.0	624	171
	PC-1-078	20.0	3.0	698	102
	PC-1-076	30.9	10.0	347	98
	PC-1-074	28.4	6.0	586	133
	PC-1-070	35.0	2.0	522	37
	PC-1-065	9.6	2.0	75	17
	PC-1-068	32.2	6.0	517	95
	PC-1-066	14.1	9.0	356	247
	PC-1-072	32.5	33.0	441	408
	PC-1-098	5.7	21.0	139	480
	PC-1-092	38.3	21.0	662	357
	PC-1-090	33.3	17.0	1468	689
	PC-1-088	22.0	16.0	391	253
	PC-1-086	38.5	16.0	850	350
	PC-1-084	21.3	7.0	403	132
	PC-1-083	26.9	9.0	436	141
	PC-1-082	27.9	16.0	702	388
	PC-1-081	15.1	10.0	347	246
	PC-1-100	34.9	20.0	698	371
	PC-1-094	41.2	10.0	919	209
	PC-1-103	21.1	5.0	573	146
	PC-1-101	49.0	3.0	531	39
	PC-1-111	28.5	6.0	460	114
	PC-1-110	34.0	6.0	689	163
	PC-1-108	28.2	3.0	392	46

	PC-1-106	27.2	4.0	316	46
	PC-1-105	19.8	6.0	411	149
	PC-1-104	25.2	6.0	275	67
	PC-1-102	5.3	4.0	98	89
	PC-1-128	42.0	22.0	641	361
	PC-1-125	12.8	18.0	356	438
	PC-1-122	3.7	18.0	78	402
	PC-1-123	23.0	13.0	534	281
	PC-1-124	30.5	14.0	725	303
	PC-1-126	17.6	22.0	375	342
	PC-1-130	41.8	30.0	586	483
	PC-1-135	39.2	36.0	1272	1291
	PC-1-136	47.3	23.0	1034	630
	PC-1-138	21.4	23.0	593	581
	PC-1-153	42.6	21.0	946	461
	PC-1-154	26.9	10.0	609	237
	PC-1-159	43.1	19.0	904	426
	PC-1-142	5.5	15.0	131	293
	PC-1-143	17.9	8.0	424	180
	PC-1-151	25.4	12.0	293	148
	PC-1-148	30.4	11.0	609	246
	PC-1-164	15.8	16.0	479	501
	PC-1-167	3.0	27.0	97	940
	PC-1-169	32.5	7.0	1241	317
	PC-1-173	11.6	28.0	418	1020
	PC-1-175	24.9	12.0	764	341
	PC-1-177	28.1	24.0	416	354
	PC-1-171	25.5	10.0	302	133
	PC-1-179	9.1	19.0	198	349
	PC-1-186	18.7	22.0	507	574
	PC-1-188	31.8	17.0	534	255
	PC-1-198	44.0	11.0	509	113
	PC-1-196	23.9	3.0	1433	169
**Maputo**	PC-1-018	16.1	20.0	323	359
**PC-B**	PC-1-002	24.6	35.0	616	856
**Count**	PC-1-006	6.7	14.0	104	212
**68**	PC-1-010	20.8	18.0	392	299
	PC-1-016	38.1	6.0	756	194
	PC-1-023	24.8	11.0	609	273
	PC-1-021	27.9	9.0	531	152
	PC-1-039	23.4	10.0	203	74
	PC-1-038	42.3	19.0	951	368
	PC-1-037	5.1	7.0	92	130
	PC-1-036	47.9	16.0	919	325
	PC-1-035	8.7	10.0	165	188
	PC-1-031	36.0	16.0	615	270
	PC-1-079	12.1	9.0	111	79
	PC-1-075	8.2	9.0	133	141
	PC-1-073	12.4	9.0	246	191
	PC-1-071	13.8	14.0	217	239
	PC-1-063	25.7	4.0	833	119
	PC-1-069	42.4	19.0	847	355
	PC-1-067	24.5	11.0	652	275
	PC-1-064	18.6	9.0	535	252

PC-1-077	18.4	21.0	239	266
PC-1-025	21.4	9.0	537	206
PC-1-033	14.0	1.0	133	13
PC-1-087	14.5	9.0	218	126
PC-1-085	24.4	10.0	593	244
PC-1-099	10.6	11.0	217	241
PC-1-095	10.5	14.0	133	145
PC-1-091	10.7	10.0	202	169
PC-1-089	7.7	12.0	141	227
PC-1-097	29.5	14.0	739	345
PC-1-093	30.3	16.0	1085	550
PC-1-107	23.6	5.0	435	116
PC-1-112	25.5	5.0	665	134
PC-1-131	8.0	14.0	113	208
PC-1-127	11.8	26.0	149	706
PC-1-132	11.5	15.0	179	277
PC-1-133	21.6	9.0	280	131
PC-1-137	19.0	16.0	503	439
PC-1-139	2.4	7.0	30	73
PC-1-140	6.9	11.0	120	199
PC-1-150	19.1	13.0	511	353
PC-1-158	36.1	17.0	804	377
PC-1-160	28.3	33.0	423	523
PC-1-144	18.1	15.0	329	288
PC-1-149	12.3	7.0	380	220
PC-1-147	5.9	22.0	109	449
PC-1-146	7.5	14.0	128	216
PC-1-163	6.1	17.0	148	403
PC-1-161	32.3	9.0	1265	375
PC-1-168	25.3	34.0	537	698
PC-1-170	21.2	29.0	434	616
PC-1-172	15.9	20.0	260	331
PC-1-174	8.8	12.0	179	275
PC-1-176	6.7	10.0	94	162
PC-1-178	28.0	23.0	802	674
PC-1-181	4.7	9.0	106	262
PC-1-182	6.6	7.0	147	163
PC-1-183	15.4	21.0	311	404
PC-1-184	21.4	16.0	317	252
PC-1-199	15.0	6.0	222	93
PC-1-197	8.9	12.0	167	252
PC-1-195	27.0	20.0	802	522
PC-1-194	25.6	20.0	425	305
PC-1-193	40.4	16.0	833	273
PC-1-192	17.0	14.0	244	182
PC-1-191	20.5	10.0	1136	542
PC-1-200	20.9	12.0	391	214
